# Towards a top-down approach for an automatic discourse analysis for Basque: Segmentation and Central Unit detection tool

**DOI:** 10.1371/journal.pone.0221639

**Published:** 2019-09-04

**Authors:** Aitziber Atutxa, Kepa Bengoetxea, Arantza Diaz de Ilarraza, Mikel Iruskieta

**Affiliations:** 1 Ixa Group, Language and Computer Systems, University of the Basque Country (UPV/EHU), Bilbao, Basque Country; 2 Ixa Group, Language and Computer Systems, University of the Basque Country (UPV/EHU), Donostia, Basque Country; 3 Ixa Group, Didactics of Language and Literatura, Bilboko Hezkuntza Fakultatea, University of the Basque Country (UPV/EHU), Leioa, Basque Country; Eberhard Karls Universitat Tubingen, GERMANY

## Abstract

Lately, discourse structure has received considerable attention due to the benefits its application offers in several NLP tasks such as opinion mining, summarization, question answering, text simplification, among others. When automatically analyzing texts, discourse parsers typically perform two different tasks: *i*) identification of basic discourse units (text segmentation) *ii*) linking discourse units by means of discourse relations, building structures such as trees or graphs. The resulting discourse structures are, in general terms, accurate at intra-sentence discourse-level relations, however they fail to capture the correct inter-sentence relations. Detecting the main discourse unit (the Central Unit) is helpful for discourse analyzers (and also for manual annotation) in improving their results in rhetorical labeling. Bearing this in mind, we set out to build the first two steps of a discourse parser following a top-down strategy: *i*) to find discourse units, *ii*) to detect the Central Unit. The final step, i.e. assigning rhetorical relations, remains to be worked on in the immediate future. In accordance with this strategy, our paper presents a tool consisting of a discourse segmenter and an automatic Central Unit detector.

## 1 Introduction

Our linguistic understanding about how to exploit the discourse properties of a text has grown in many ways, as described by [1]. Discourse parsing is a very challenging task and several authors have shown that discourse structure is crucial in obtaining a better understanding of texts. Exploiting discourse structure information adequately could be the key to improving different NLP tasks such as: *i*) summarization [2], *ii*) complex question answering [3] *iii*) opinion mining [4] and sentiment analysis [5–7].

Our approach to discourse here follows Rhetorical Structure Theory (RST) [8], a discourse theory that describes coherence of a text with rhetorical relations between text-spans forming a hierarchical discourse tree (RS-tree). Elementary Discourse Units (EDU) are minimal text-spans of a discourse tree. By linking these EDUs and following an incremental, modular strategy, all spans of a coherent text have their own function in the RS-tree. There are two kinds of coherent relations, symmetric (or paratactic) and asymmetric (hypotactic) discourse relations. Symmetric relations are also known as multinuclear relations, for example, LIST, SEQUENCE or CONTRAST, because they have more than one nucleus, and asymmetric relations are called nuclear relations, because they have one nucleus and one satellite (or the semantically modified text-span) relation, for example, ENABLEMENT, CONCESSION, SUMMARY, ELABORATION, CAUSE or PURPOSE. The nucleus text-span is the most relevant span concerning the writer’s purpose. Almost every relation is recursive, except for the Central Unit (CU) which is the most salient text-span of the RS-tree [9]. Although the CU is not always indicated (as sometimes the main topic can be elided), we agree with [10] and [11] in that most of the time the CU can be detected. This has been shown in different corpora and different languages [12–14]. It is noteworthy that, as [9] stated, there are texts with multiple CUs in the RST Basque Treebank: 23,33% (14 of 60 texts). This happens because the main idea is expressed in different clauses or sentences and can be linked with multinuclear relations.

Practical benefits have been driving studies aiming to develop an Automatic Discourse Analyzer (ADA) under different discourse theories. These are some freely available and testable ADAs for English: *i*) [15–18] developed parsers (to cite some) under Rhetorical Structure Theory (RST) [8].

*ii*) [19] chose the Segmented Discourse Representation Theory (SDRT) [20] to build the ADA (http://gmb.let.rug.nl/webdemo/demo.php). *iii*) [21] followed Penn Discourse Treebank (PDTB) style [22] (http://wing.comp.nus.edu.sg/~linzihen/parser/).

The quality of these supervised parsers relies heavily on the size of corpora employed and the quality of the manual annotation of these corpora, which is both difficult and expensive. Typically, discourse-structure parsers follow two steps: discourse segmentation and relation detection. Currently there are two online parsers for two different languages available for testing. One is the previously mentioned parser developed by [17] and the other, DiZer, which was developed by [23] for Brazilian Portuguese.

Nevertheless, research has been oriented towards building partial parsers for RST which do not complete all the phases of an automatic discourse analyzer but do complete some stages of discourse parsing. The output obtained from a partial parser, if accurate, it is useful in other NLP tasks where an entire discourse tree is not required. [24] claim that the best strategy for building an RS-tree is to start by detecting the intra-sentential relations, for two reasons. On one hand, both choices and ambiguity are fewer than in the inter-sentential relations, and on the other, some intra-sentential discourse structures can be derived from syntactic information.

Results from the different RST parsers give clear proof of this fact, and all, [17, 25, 26], obtained better results for intra-sentential relations than for inter-sentential relations; [27] developed a segmenter and intra-sentential RST parser for Spanish (http://diseg2.termwatch.es/). [28] built a segmenter (http://ixa2.si.ehu.es/EusEduSeg/EusEduSeg.pl) and [29] a CU detector (http://ixa2.si.ehu.es/CU-detector/) for Basque. [30] developed a segmenter for German.

[17] measured precision, recall and F-score over rhetorical relations on their parser (among other features), employing the quantitative evaluation method [31]. The parser is available online and they reported the best results to date in all three measurements at intra-sentential level. Following the same reasoning, [32] show that the lack of agreement at inter-sentential discourse level, is greater not only in the relation tags, but also in the relation attachment locus. The qualitative evaluation method [33]) employed in this project describes the types of agreement over segmentation, attachment, composition, nuclearity and relations. This evaluation method highlights relevant aspects, such as the critical role a relation attachment locus plays in correctly annotating the relation’s label. Thus, some disagreements in relations are a consequence of a lack of agreements in the attachment locus which happens to be greater at inter-sentential level.

As part of building a whole parser, we propose a top-down strategy, integrating, in a first stage, a discourse segmenter and an automatic Central Unit detector, and leaving as the next step the identification of discourse relations between discourse segments. In our opinion, including Central Unit (CU) identification in the top-down strategy proposed, will facilitate the decision of where to attach some inter-sentential relations. [9] pinpoint Central Unit identification as a key step in the manual annotation of relational structure. Identifying in advance which the CU is, increases inter-annotator agreement in the process of building RS-trees. Our proposal is based on the idea that an automatic processing strategy should follow manual practices performed by human annotators, principally where they have been empirically shown to be reliable.

Therefore, with the future objective of developing a complete discourse parser, this work aims to build and evaluate automatic discourse segmentation and Central Unit detector based on neural networks, in order to use this partial parser in different NLP tasks: *i*) summarization [2], *ii*) complex question answering [3] *iii*) opinion mining [4] and sentiment analysis [5–7] *iv*) evaluation of scholars’ summaries [34].

To explain what a CU is, we first need to define what an Elementary Discourse Unit (EDU) is. Nowadays, the definition of an EDU is controversial even in RST [35], because it depends on granularity, and several granularity measures have been proposed within RST. In this paper, we will consider discourse units as functionally independent units or clauses [36]. There are three types of subordinate clauses that can be distinguished: *i*) complements (which function as noun phrases), *ii*) relative clauses (which function as noun modifiers) and *iii*) adverbial clauses (which function as modifiers of verb phrases or entire clauses). [37] stated that some subordinated clauses, for example, adverbial clauses, can be seen as clause linkages, because it is the adverbial clause which provides a (discourse) thematic role to the main clause. For more information on adverbial clauses, refer to [38, 39].

Our segmentation guidelines follow [40] and they were implemented for Basque in [28] in the form of rules.

The CU of an RS-tree, is the clause (or EDU) which best expresses the topic or the main idea of a text. The CU can be a single EDU, or a group of EDUs, because in RST there are various paratactic relations which connect EDUs at the same level and thus cover the entire structure of the text. Other groups of EDUs (spans) are linked to it, but the CU is not linked to any other unit and, therefore, no other nuclei of the RS-tree have the same degree of central importance [41] as the CU. The CU is similar to the thesis statement defined by [11], but in contrast to this thesis statement, which can be elided, in an RS-tree there will always be at least one EDU that is not linked to another unit. In those cases we determined how to choose the CU following [9].

**Fig 1:**
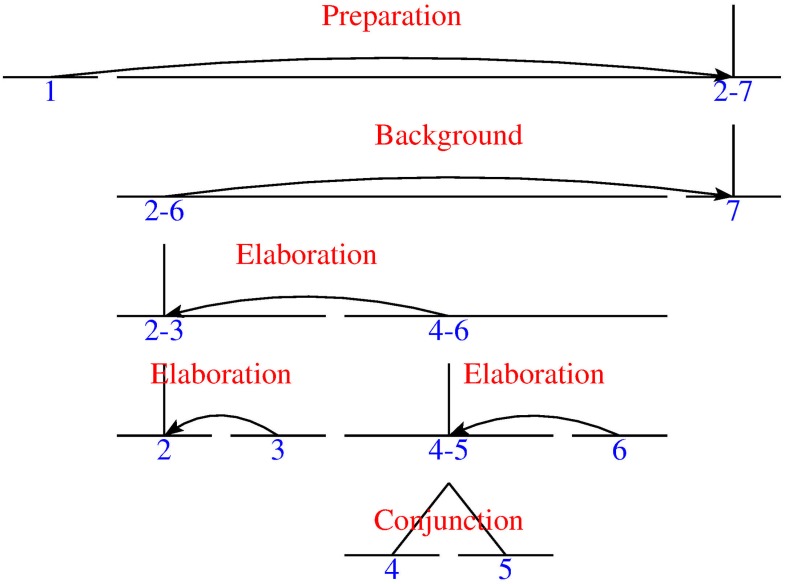
An RS-tree of GMB0301.

Usually, writers unambiguously express which the CU is by using several indicators or languages forms. Fig 1 shows a segmentation example. The original text in Basque of GMB0301 is:

[Estomatitis Aftosa Recurrente (I): Epidemiologia, etiopatogenia eta aspektu klinikopatologikoak.]_1_ [“Estomatitis aftosa recurrente” deritzon patologia, ahoan agertzen den ugarienetako bat da,]_2_ [tamainu, kokapena eta iraunkortasuna aldakorra izanik.]_3_ [Honen etiologia eztabaidagarria da.]_4_ [Ultzera mingarri batzu bezala agertzen da,]_5_ [hauek periodikoki beragertzen dira.]_6_ [Lan honetan patologia arrunt honetan ezaugarri epidemiologiko, etiopatogeniko eta klinikopatologiko garrantsitsuenak analizatzen ditugu.]_7_

(1) [Recurrent aphthous stomatitis (I): epidemiologic, etiologic and clinical features.]_1_
  [Recurrent aphthous stomatitis is one of the most frequent oral pathology.]_2_ [It has a controversial etiology.]_3_ [It is characterised by the apparition of painful and recurrent ulcers,]_4_ [that has a variable size, location and duration.]_5_ [These ulcers appear periodically.]_6_ [In this paper we analyze the most important epidemiological, etiological, pathological and clinical features of this common oral pathology.]_7_

Once the text is segmented, as in Example (1), the next step consists of identifying indicators to find the Central Unit of this text: *i*) *In this paper*, the demonstrative *this* and the noun *paper* refers to the work the writers are presenting. *ii*) The superlative *the most* and the adjective *important* indicate that this sentence is prominent in the text. *iii*) The verb *analyze* is a common verb for expressing the main action of piece of research [9]. Its meaning is associated with the WordNet Synset *analyze_1_*, which belongs to the reasoning category determined by the SUMO ontology. *iv*) The pronoun *we* indicates an action or the topic performed by the writers. All these indicators and others will be transformed into features to automatically detect the Central Unit.

After identifying the CU, constructing the RS-tree of the Example (1), which is presented in Fig 1, becomes easier. In Fig 1 showing the EDU_7−7_, the CU is the nucleus which has no satellite above it and its sole parent is the span_2−7_ which is not attached to any other EDU or span:

*i*) EDU_1−1_ is attached to span_2−7_.
*ii*) The parent of the EDU_2−2_, which is the span_2−6_, is attached to EDU_7−7_.
*iii*) EDU_3−3_ is linked to span EDU_2−2_.
*iv*) The parent of EDU_4−4_ and EDU_5−5_, which is the span_4−6_, is attached to span_2−3_.
*v*) EDU_6−6_ is attached to span_4−5_.

These are the manual annotation steps and, as stated above, finding the CU automatically after segmentation will be helpful for discourse parsers to decide the attachment of some inter-sentential relations (where there is less precision). This is especially true in domains with a fixed discourse structure, and in genres or domains that do not follow newspaper macro-structure, where the CU is at the beginning of the text. Although this is an interesting discussion, it falls outside the scope of this paper. If the parser knows in advance that the CU is EDU_7−7_ in Fig 1 it will attach the span_2−6_, if it has this span, to EDU_7−7_ using a S-N order relation, for example a BACKGROUND relation, following an incremental, modular annotation strategy.

**Fig 2:**
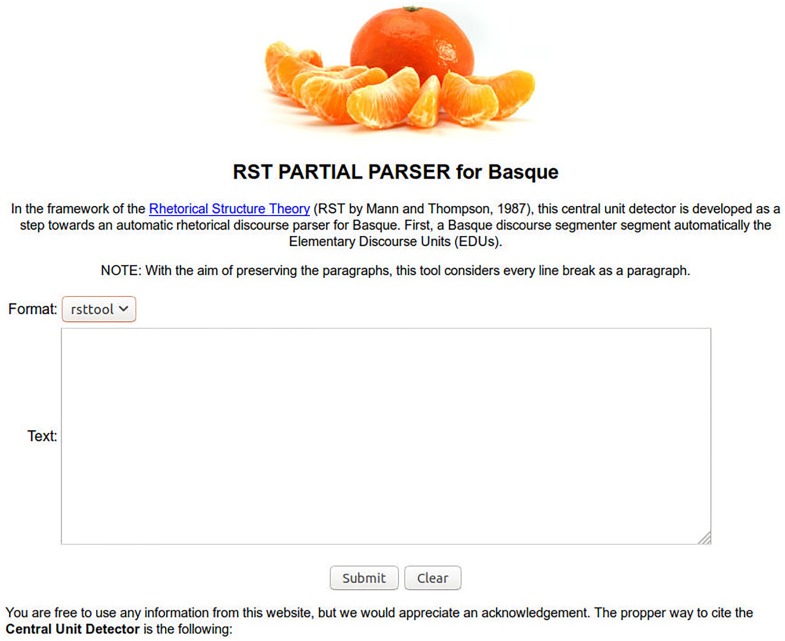
The partial parser.

The aim of this paper is to present a tool that segments plain text and detects the CU using deep-learning and several other machine-learning techniques to improve previous results obtained in such tasks. In our case, identifying the CU will be especially useful in the future in two tasks we are planning to pursue shortly: *a*) advanced NLP applications (question answering, summarization and sentiment analysis) for the Basque language and *b*) manual RST annotation. To do so, we followed the theoretical principles of RST for both tasks: *i*) segmentation [42] and *ii*) CU detection task [43]. Regarding segmentation, we have used neural networks with a result of 0.85 F_1_ in the test set and, for CU detection, we have essayed with Bernoulli Naive Bayes (BNB) system with Linguistic Features (LF), 1-CNN with pre-trained word embeddings and a Logistic Regression model with BoW approach and an ensemble system. The best CU detector is the ensemble system with 0.607 F_1_ in the test set. We have also presented an original set of experiments studying the effect of using the segmenter output as input for the Basque CU detector, obtaining the best result with the ensemble system, 0.592 F_1_ in testing. These results outperform previously obtained results in these tasks in Basque, for which a demo can be tested as shown in Fig 2.

The remainder of this paper is structured as follows. Section 2 lays out related work and the theoretical framework and Section 3 shows the methodology used to build the CU detector. Section 4 presents the system and Section 5 sets out the results of the detector. Finally, Section 6 will be devoted to discussion and section 7 to results and future work.

## 2 Related work

Until now, segmentation and the CU detection tasks were isolated tasks and CU detection was performed on a manually segmented corpus. This work presents a unique tool that accomplishes automatic segmentation and CU detection using deep-learning and other machine-learning techniques.

### 2.1 The automatic discourse segmenter

There are several ways of pursuing the automatic segmentation task; using rule based techniques as in: *i*) [28] for Basque, *ii*) [44] for Spanish, and *iii*) [40] for English. Using machine-learning techniques, for example, perceptron, as in [45] for French. The segmentation projects mentioned for Spanish, French and English obtained F-measures (F_1_) ranging from 73% to 85%.

Both perceptron and rule-based systems require heavy feature engineering work in order to find the right feature-context combination. The latest segmentation projects, more precisely the ones participating in the recently organized DISRPT 2019 Shared Task on automatic discourse unit segmentation and connective detection [46], employ neural network techniques in the same way this work does. Results of the DISRPT 2019 Shared Task can be seen in Table 1.

**Table 1:** EDU segmentation results on Basque treebanked data in ACL discourse Shared Task 2019. IXA corresponds to our team.

System	ToNy			GumDrop			DFKI RF			IXA		
	P	R	F_1_	P	R	F_1_	P	R	F_1_	P	R	F_1_
eus.rst.ert	89.77	82.87	**86.18**	90.89	74.03	81.60	92.77	60.54	73.27	91.19	80.27	**85.38**

Several teams participated in the DISRPT 2019. Among the best proposals to mention some:

- Tony [47] employ single-layer bi-directional LSTM models with different pre-trained embeddings, and they get the best results using contextual embeddings.
- DFKI RF [48] uses a Random Forest (based on Scikit-learn [49] whose input is a combination of dependency tree and constituency syntax information. In addition, they use a LSTM-based method (based on Keras [50]) with pre-trained word embeddings [51].
- GumDrop segmenter [52] is an ensemble of 3 modules: a) The sub-tree module focuses on dependency sub-graphs, looking at a trigram around the potential split point. b) The BoW-Counter module, which predicts the number of segments in each sentence using Ridge regressor with regularization. c) NCRF++ [53], a bi-LSTM/CNN-CRF sequence labeling framework and FastText embeddings. Predictions from these 3 different machine-learning approaches are all fed to a “meta-learner” or blender classifier.

In DISRPT 2019, our group (IXA) used a BiLSTM+CRF [54] to build our segmenter. These kinds of systems allow to avoid all the feature engineering process, since the BiLSTM neural network itself, through its gates, learns the right feature-context configuration. Our segmenter uses both, syntactic-semantic (word embedding) and purely morphosyntactic information (POS and case or complementizer mark), following a form-function approach. Circularity is avoided in the annotation process: there is no rhetorical constraint when segmenting the text. Note that as [40] we kept aside “same-unit” constructions.

The present work, compared to our DISRPT 2019 participation, although based on the same BiLSTM+CRF architecture, applies different features. We will show in section 5 that our current segmenter obtains a 12-point improvement (30 points regarding the intra-sentential segmentation) compared to our previous rule-based segmenter. It also improves the results of our DISRPT 2019 Shared Task segmenter.

### 2.2 The Central Unit detector

Several CU detectors have been developed based on manual segmentation for different languages, genres and domains:

- For Basque, in [13, 55] the CU was detected using rule-based methods obtaining the best F-score 0.512 in the test dataset. In [56] the CU was identified by keywords and some lexical-syntactic patterns using a Bernoulli Naive Bayes (BNB) classification model. After using hill-climbing wrapper method [56] obtained the best F-score 0.57 in the test dataset for Basque, choosing nouns, verbs, bonus (some adverbs and adjectives), determinants, pronouns, segment position, title words, auxiliary verbs and 3 combinations (nouns + determinants, pronouns + nouns and y verbs + auxiliary verbs) as feature set. The corpus was built compiling 100 scientific abstract texts. The scientific abstract texts belonged to the following 5 domains: Medicine, Terminology, Science, Health and Life.
- For Spanish, in [57] the CU was identified by Bag-of-Words (BoW), EDU position and title word occurrence information using Multinomial Naive Bayes (MNB) and Sequential Minimal Optimization (SMO) classification models. SMO classification model was the best model, obtaining an F-score 0.806 in the 10-fold cross-validation and F-score 0.759 in the test dataset. The gold standard was created with 73 abstract texts. The corpus belonged to the following two domains: Psychology and Linguistics.
- For Brazilian Portuguese, in [13] the CU was detected using rule-based methods obtaining the best F-score 0.553 in the test set. In [14] the CU was identified by using linguistic features defined by [58] and automatic features (BoW and chi-squared statistics to select features) with EDU position and title-word occurrence information in Multinomial Naive Bayes (MNB), Bernoulli Naive Bayes (BNB) and Sequential Minimal Optimization (SMO) classification models. The SMO classification model with linguistic features obtained the best classification result, F-score 0.76 in the 10-fold cross-validation and F-score 0.657 in the test set. The gold standard was created with 100 argumentative answer texts written by candidates for the Summer 2013 entrance exams at the Universidade Estadual de Maringa (UEM).

In this work, we present several CU detectors using machine-learning and deep-learning techniques on a corpus of 140 scientific abstract texts belonging to the following 7 domains: Medicine, Terminology, Science, Health, Life, Economy and Computer science. Although CU has genre and domain constraints and we have added two new domains, we have improved the results of the CU detector obtained by [56].

The double sequential task of this work, therefore, is similar to [28, 40] in segmentation and similar to [11, 56, 59] in the detection of the CU. To our best knowledge, this proposal is the first to unify these two steps automatically.

## 3 Methods

### 3.1 Corpus

As mentioned before, the corpus used for CU detection contains 2,998 EDUs and 140 scientific abstract texts belonging to 7 domains. A more detailed description is presented in Table 2.

**Table 2:** Corpus description: Domains, sources and measures.

Domain	Source	Texts	Words	EDUs	CUs
Medicine	Gaceta Médica de Bilbao, 2000-2008	20	1,941	283	31
Terminology	Int. Conference on Terminology, 1997, UZEI	20	3,242	584	39
Science	Scient. articles, Faculty of Science, UPV/EHU	20	3,735	603	28
Health	2nd Symp. of Basque Researches,2014, UEU	20	3,156	487	22
Life	1st Symp. of Basque Researches, 2010, UEU	20	3,598	592	23
Economy	Uztaro Journal, UEU	20	1,394	216	25
Computer science	Ekaia Journal, UPV/EHU	20	1,440	233	24
**Total**		140	18,506	2,998	192

This corpus, compared to the one used by [28, 55, 56] for Basque, contains 40 additional texts, as we included 2 new domains (economy and computer science). The size—140 texts—is similar to or larger than others created for similar aims, such as [40] (9 texts) and [44] (20 texts) for segmentation, and [60] (32 texts) and [11] (100 texts) for CU detection. The corpus in Table 2 was randomly divided into 3 non-overlapping datasets: 84 texts as the training set, 28 texts as the development set and 28 texts as the test set (Table 3).

**Table 3:** Corpus for the CU.

Dataset	Texts	Words	EDUs	CUs	CU difficulty
**Train**	84	10,668	1,782	116	0.0651
**Dev**	28	4,118	631	41	0.0649
**Test**	28	3,720	585	35	0.0598

The task’s difficulty to find the CU has been calculated as follows: $Difficulty=\frac{CUs}{EDUs}$, where the closer it is to 1 the easier it is to determine the CU.

All the experiments were done on the development set, leaving the best systems for the final test.

For the segmentation task, and exclusively for segmentation training purposes, we added 335 new texts with 8,633 EDUs (see Table 4) to the 84 training texts used to train the CU detector (see Table 3). The 335 new texts belong to different genres and domains and are not annotated with CUs. The development and test sets are the same as those employed in the CU task (see Table 3).

**Table 4:** Corpus for segmentation.

Dataset	Texts	Words	EDUs
**Train**	84+335	110,841	10,415
**Dev**	28	4,118	631
**Test**	28	3,720	585

The whole corpus was syntactically parsed in order to obtain some morphosyntactic features such as POS, case and sentence complementizers. We applied two different dependency parsers. This allowed us to build different segmenters depending on the source of the syntactic information feeding the biLSTM+CRF network. The rationale behind this decision was to measure the impact one might expect syntax to have on segmentation. The two parsers were Maltixa [61], explicitly built for Basque, and a language-agnostic parser, UDPipe [62], trained on the Basque UDTreebank [63].

### 3.2 Annotation reliability

The full corpus was annotated by two linguists who were familiar with the RST, using the RSTTool [64].

The annotation phases were the following:

*i*) Annotators segmented the texts manually following [42].
*ii*) For each of the 140 texts in the CU corpus subset, both annotators identified the CU in [9].
*iii*) The results were evaluated and harmonized following [42].

### 3.3 CU agreement between annotators

Two annotators manually recognized the CUs. The agreement between the annotator-1 (A1) and the annotator-2 (A2) using Kappa coefficient [65] was 0.798 in the training set (out of a total of 1.782 EDUs), 0.775 in the development set (out of a total of 631 EDUs) and 0.802 in the test set (out of a total of 585 EDUs) respectively. This consensus (between the values 0.61–0.8) indicates a substantial agreement according to [66].

### 3.4 System evaluation measures

Regarding the evaluation of the segmenter, the usual IBO tags were employed to annotate corpus segments; so every segment starts with a B-SEG tag and any segment’s internal word is tagged as I-SEG until a sentence boundary or the beginning of another segment is found. B-SEG is the most informative tag, and therefore, in order to evaluate the performance of the segmenter we employed the usual precision (Prec.), recall (Rec.) and F-score (F_1_) metrics over the B-SEG tags, measuring both the performance over all B-SEG tags, and exclusively over the intra-sentential ones, since these are the most difficult to capture.

We evaluated the CU detector by means of the same metrics. To assess the results of the CU detector on the output of the segmenter we have used an exact-match scenario (matching only segments that have the same automatic and gold beginning segment label (B-SEG)). For example, exact-match precision is calculated as the number of correct CUs divided by the total number of CUs proposed by the system, but only taking into account the segments that start with the same gold token.

## 4 The system

### 4.1 Pre-trained word embeddings

[67] studied the role of context and dimension on the effectiveness of different word embeddings for different language processing tasks. These tasks ranged from more syntax-related (dependency parsing, NER) to more semantics-related tasks (co-reference and sentiment analysis). They concluded that it is crucial to choose the right kind of embeddings to get the best results on specific tasks. Following [67], under the same premise as that stated above, regarding the application of two distinct parsers, we found it relevant to measure the impact different word representation might have on the segmentation task. For that matter, we tested two types of word embeddings.

On one hand, Elhuyar Basque word embeddings (our embeddings) calculated on Elhuyar web Corpus [68] using gensim’s [69] word2vec skip-gram [70], with a dimensionality of 350 and using a window size of 5. The Elhuyar web corpus was automatically collected by scraping the web, and it contains around 124 million word forms. On the other hand, we also employed 300-dimensional standard out-of-the-box Facebook’s FastText [71] embeddings.

### 4.2 Discourse segmentation

In the lines of work done using neural networks to pursue chunking, NER, POS tagging [54] we carried out the discourse segmentation phase in two steps following the form-function approach:

1. Obtaining information for each word to use it later as input for BiLSTM+CRF, more precisely: *a*) Word embedding. *b*) POS and case or subordination mark if the word has any (see Section 3.1).
2. Performing the actual segmentation built on a BiLSTM+CRF system.

Instead of initializing the embedding layer with randomly selected values, we employed the aforementioned pre-trained word embeddings, as described in Subsection 4.1. The case and subordination mark associated with each word comes from the parser’s output (either MaltParser’s Basque version Maltixa [61] or the UDPipe). Table 5 shows the input for training the segmenter. Maltixa POS tags used in Table 5: IZE (noun), ADI (verb), PUNT (punctuation), ABS (absolutive), GEL (gelative), ERG (ergative), GEN (genitive), ALA (ablative).

**Table 5:** A training example sentence of BIZ04.

wordForm	Translation	POS	CASE	SegTag
Ernalketa	fertilization	IZE	ABS	BSEG
gertatzeko	occur	ADI	GEL	ISEG
espermatozoideek	sperm	IZE	ERG	BSEG
emearen	female	IZE	GEN	ISEG
umetoki-tronpara	uterine tube	IZE	ALA	ISEG
heldu	arrive	ADI	_	ISEG
behar_dute	(they) need	ADI	_	ISEG
.		PUNT	_	O

**Fig 3:**
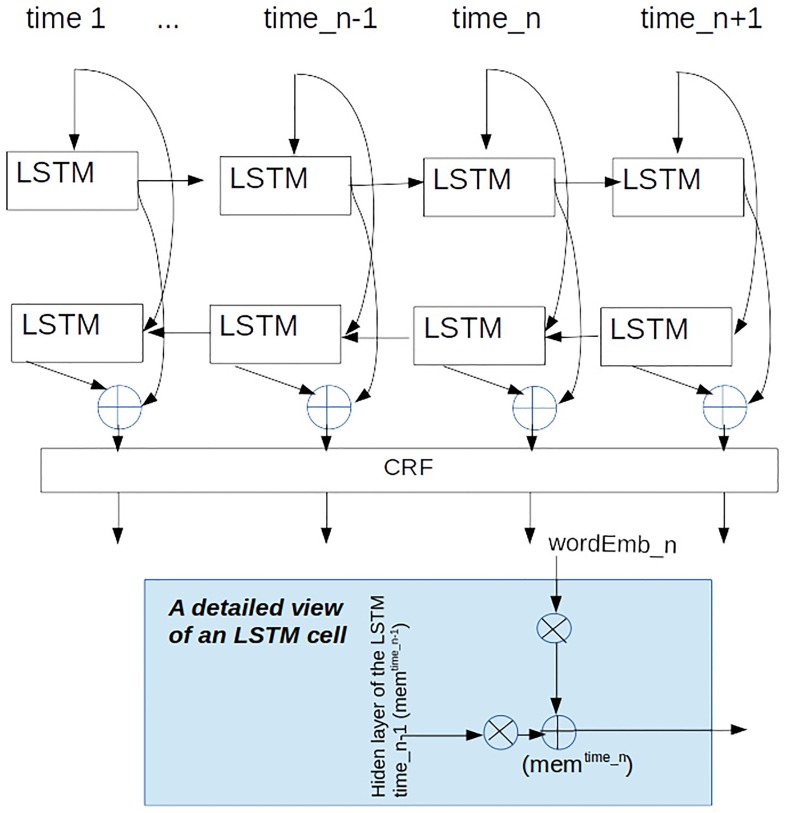
Graphical view of the segmenter.

LSTM [72] neural networks are widely used for sequential labeling where the input-output correspondence depends on previously treated elements. This dependency is accomplished, at each time step, in the corresponding LSTM cell by feeding each hidden state with the output of the previous hidden state, as shown in Fig 3. So, the segmentation process consists of taking an input sequence (*x*_1_, *x*_2_, *x*_3_, ⋯, *x*_*n*_) and obtaining the corresponding segmentation tag output (*h*_1_, *h*_2_, *h*_3_, ⋯, *h*_*n*_) at each step, bearing in mind not only information about the current input word, but also about the previously treated input. Contrary to other sequence-to-sequence algorithms (perceptron [45]), LSTMs are able to automatically learn which context needs to be remembered or forgotten to pursue the tagging. Bi-LSTMs are a special case of LSTM, where two LSTM nets are employed; one treating the input sequence from left to right (forward LSTM) and the other from right to left (backward LSTM).

For this work we took as our point of reference the implementation done by [54], adapting it to accept not only the embeddings, but also additional information like POS or case and syntactic subordination information at each step. The equations below formally describe a memory cell in this implementation:

$$\begin{array}{ccc} i_{t} & = & \sigma (W_{x_{i}}x_{t}+W_{h_{i}}h_{t-1}+W_{c_{i}}c_{t-1}+b_{i}) \end{array}$$ (1)

$$\begin{array}{ccc} {\tilde{c}}_{t} & = & \tanh(W_{x_{c}}x_{t}+W_{h_{c}}h_{t-1}+W_{c_{i}}c_{t-1}+b_{c}) \end{array}$$ (2)

$$\begin{array}{ccc} c_{t} & = & \left(1-i_{t}\right)⊙c_{t-1}+i_{t}⊙{\tilde{c}}_{t} \end{array}$$ (3)

$$\begin{array}{ccc} o_{t} & = & \sigma (W_{x_{o}}x_{t}+W_{h_{o}}h_{t-1}+W_{c_{o}}c_{t}+b_{o}) \end{array}$$ (4)

$$\begin{array}{ccc} h_{t} & = & o_{t}⊙\tanh\left(c_{t}\right) \end{array}$$ (5)

- *σ* and tanh represent the sigmoid and hyperbolic tangent, respectively, which introduce nonlinearities in the network, thus increasing the predictive power of the network.
- *t* and *t* − 1 correspond to the current and previous time steps, respectively.
- *c*_*t*_ defines the current state of the memory cell by taking into account how much of the previous state cell should be forgotten ((1 − *i*_*t*_) ⊙ *c*_*t* − 1_) and how much information will be updated ($i_{t}⊙{\tilde{c}}_{t}$).
- *i*_*t*_ represents which values will be updated and ${\tilde{c}}_{t}$ represents which new candidates could be added to the state.
- *o*_*t*_ defines, through the sigmoid (*σ*), which part of the information stored in the cell will become output.
- *h*_*t*_ corresponds to the hidden state. In this case, and as it is a Bi-LSTM, *h*_*t*_ will be calculated as the concatenation of both contexts (right to left $\vec{h_{t}}$ and left to right $\overset{\leftarrow }{h_{t}}$).

### 4.3 Central Unit detection

#### 4.3.1 Single systems

The CU detector performed as follows, using different standard baseline methods such as Bernoulli Naive Bayes (BNB), Logistic Regression (LR) and one-layer Convolutional Neural Networks (1-CNN), and different features such as Linguistic Features (LF), Bag of Words (BoW) with tf-idf model and word embeddings:

- As our baseline, we have used the best system [56]: CU is identified by keywords and some lexical-syntactic patterns using a Bernoulli Naive Bayes (BNB) classification model. The BNB approach is a classic naive Bayes variant. BNB trains classifiers in the absence and presence of indicators or features, and using this information we can build a model to classify or select from a text the EDU that is the most likely candidate to be labeled as CU. After using the hill-climbing wrapper method the best feature set was: a list of nouns and verbs and a bonus of some adverbs and adjectives, some determinants, first person pronouns, segment position, title words, first person auxiliary verbs and 3 combinations (nouns + determinants, pronouns + nouns and verbs + auxiliary verbs).
- One-layer CNN (1-CNN) model with pre-trained word embeddings: We have implemented a model similar to [73]. After an optimization process similar to [74], we have used: rectified linear units, filter windows of 2, 3, 4 with 100 feature maps each, dropout rate of 0.5, l2 constraint of 3, 1-max pooling. The training is done through Stochastic Gradient Descent (SGD) over the full training set with the Adadelta update rule [75], with pre-trained word-embeddings and finally we have used the softmax function to select the CU with the highest probability in a text. These values were chosen via a grid search on the development set. We do not otherwise perform any dataset-specific tuning other than early stopping on development set.
- Logistic Regression (LR) [49] system with Bag of Words (BoW): LR is a learning algorithm used in a supervised learning problem when the output is all either zero or one. The goal of LR is to minimize the error between its predictions and training data. Given a segment represented by a feature vector, the algorithm will evaluate the probability of that segment as a CU. To detect the best features automatically, we performed the following steps:
  - We converted all words to lower case.
  - We converted segments into a feature vectors using a TF-IDF [76] BoW model. To limit the size of the feature vectors, we used different sizes (500, 800, 1000, 2500, 5000 and 15000) of most frequent words including unigrams, bigrams and trigrams. Finally we performed the experiment using 800 words in LR.
  - We also added EDU position and title-word occurrence information to the feature vector.
  - We applied an automatic feature selection which is a classic refinement method in classification. It is an effective dimensionality reduction technique to remove noise features. In general, the basic idea is to search through all possible combinations of attributes in the data to find which subset of features works best for prediction. Removal is usually based on some statistical measures, such as segment frequency, information-gain, chi-square or mutual information. In this research, we tested the two most effective feature selection methods: *i*) chi-square and *ii*) information-gain using different sets of attributes: 50, 100, 450 and 1000. Finally, we performed the experiment using chi-square with a set of 450 words in LR.

#### 4.3.2 Unweighted voting algorithm for ensemble of classifiers

In this paper, we explored the advantages of using a simple unweighted voting system to create an ensemble from the three base-level classifiers. With the unweighted voting system, the predictions of the base-level classifiers are added up for each class, and the class with the highest number of votes determines the prediction for the ensemble [77].

The quality of the combined system depends on the precision and the diversity of the base-level classifiers [78]. Given 3 classifiers *h*_1_, *h*_2_, *h*_3_ and ‘x’ being new data to be classified, if all systems were similar, when one of them *h*_1_ (x) gave an error, the rest would also show it. However, if the classifiers are sufficiently diverse, even if *h*_1_ (x) were wrong, then *h*_2_ (x) and *h*_3_ (x) could be correct, and then, if done by majority vote, the combined set would correctly classify the data ‘x’. For the ensemble system to classify a segment as a CU, the vote of at least two of the classifiers is necessary.

The use of this ensemble system overcomes the problem of over-fitting due to the small amount of training data.

To increase the quality and diversity of the ensemble system, we used different systems with different features in each system. While indicators were used in the BNB system, pre-trained word-embeddings were used in the 1-CNN system, and the BoW approach was used in conjunction with the LR model that does not take ordering into account.

#### 4.3.3 Post-process

Our system has a module to select at least one CU per text when the systems classify all the segments of a text as non-CU. Depending on the classifier, we can apply different techniques to select at least one CU. In the case of BNB, CNN and LR, the classifiers always return the probability of an EDU to be labeled as CU. So the module uses this value to select at least the most likely EDU to be labeled as CU. In the case of ensemble systems, we combined the 3 simple systems with each post-process stage, but when the ensemble system selects all the EDUs as non-CU, the decision of the BNB system with post-processing is chosen as CU. We selected the BNB system with post-processing after experimenting with the 3 simple systems with a post-processing stage on the development set.

## 5 Results

### 5.1 EDU segmentation

Table 6 shows segmentation task results. First, it shows the results of a previously implemented rule-based segmentation system [28]. As [28] reported, their first segmenter for Basque checks if there is an adjunct verb in both sides of a comma or a conjunction and uses 6 other rules to detect subordinate clauses such as temporal, causal, concessive, conditional and purpose.

**Table 6:** Results of the previous rule based system and of the current Bi-LSTM+CRF segmenter.

			General results			Intra-sentential level		
System	Data	Acc	P	R	F_1_	IntS_P	IntS_R	IntS_F_1_
RuleBased	Dev	.092	.086	.068	.076	.056	.030	.039
	Test	.091	.088	.063	.073	.062	.027	.038
Auto(Malt+OurEmb)	Dev	.098	.092	.089	.091	.084	.077	.080
	Test	.098	.091	.085	.087	.079	.067	.072
Auto(Malt+FastTextEmb)	Dev	.098	.090	.084	.087	.078	.065	.071
	Test	.098	.091	.083	.087	.078	.062	.069
Auto(UD+OurEmb)	Dev	.097	.089	.077	.082	.073	.049	.060
	Test	.097	.088	.078	.083	.070	.05	.058
Auto2/3(Malt+OurEmb)	Dev	.098	.089	.083	.086	.075	.063	.069
	Test	.097	.089	.082	.085	.074	.060	.066

Accuracy(Acc) over all IBO tags. Precision (P), Recall (R) and F-score (F_1_) correspond to the beginning of the segments (B-SEG). Intra-Sentence (IntS) refers to non-sentence initial B-SEGs.

The table then proceeds to report the results of different segmenters built varying the parser (Malt or UDPipe) and the embeddings (our embeddings or FastText’s) employed to obtain input for the BiLSTM+CRF neural network. As explained in section 4.2, the segmenter input for each word is composed of the embedded word, its POS, case and syntactic dependency relation. In Malt+OurEmb the input corresponds to the POS, case and syntactic dependency provided by the Maltparser, while the embeddings are the ones we calculated using the Elhuyar web corpus. Malt+FastTextEmb diverges from Malt+OurEmb in that the embeddings correspond to those of FastText. And finally, in UD+OurEmb, unlike Malt+OurEmb, the POS, case and syntactic dependency relation were obtained by means of the UDpipe parser.

We applied the typical random split data to train, develop and test, using 60%, 20% and 20%, respectively (see 3.1). Regarding the accuracy, although all systems obtain results over 0.9, the biLSTM+CRF segmenters reach almost 100%, while the rule-based system hardly improves over 90%. In all cases, accuracy on the test set is slightly lower than on the development set.

Regarding Precision, Recall and F-score, results show that all BiLSMT+CRF improve in all measures with respect to the previous rule-based system. As expected, the improvement is greater in terms of recall than in terms of precision, and especially in the intra-sentential measures. The 33-point increase in intra-sentential recall which BiLSTM+CRF systems score on average, pushes the F-score value of these segmenters to 31 points and 29 points on average in both development and test folds respectively for intra-sentential segments, even if the size of the training corpus is quite small compared to the size of the corpora usually employed with neural networks.

Concerning the effect syntax might have on segmentation, Malt+OurEmb overcomes UD+OurEmb in 20 and 14 F-score points in the development and test folds respectively. Finally, different word representations also show an impact on segmentation, and in conclusion, we found that by using our embeddings (Malt+OurEmb) we got better results (more that 9 and 3 F-score points in development and test sets respectively) than using FastText embeddings for Basque (Malt+FastText).

In all combinations, Malt+OurEmb obtained the best results. Therefore, we chose it to carry out the segmentation to be the input for the CU detector. To this end, we split the training folds in three folds to segment it by means of cross-validation. Development and test sets, where segmented, used the best form of the three cross-validation models. Auto2/3(Malt+OurEmb) shows the results.

### 5.2 Central Unit detection

First, we analyze the results using as input segmentation gold standard tags (Gold) obtained from the Basque RST Treebank [79]. Table 7 shows the results of applying 4 different systems BNB with Linguistic Features (LF), 1-CNN with word embeddings, LR with BoW and an Ensemble system without any post-process (-) or with post-process (+).

**Table 7:** CU result’s obtained from the gold segmentation(Gold) without any post-process (-) or with post-process (+).

System	Post	Data	C	E	M	P	R	F_1_
Human	-	Dev	26	15	15	0.634	0.634	0.634
		Test	31	7	4	0.815	0.885	0.849
BNB	-	Dev	24	58	17	0.292	0.585	0.390
		Test	22	21	13	0.511	0.628	0.564
	+	Dev	24	61	17	0.282	0.585	0.380
		Test	22	28	13	0.440	0.628	0.517
1-CNN	-	Dev	7	8	34	0.466	0.170	0.250
		Test	12	4	23	0.750	0.342	0.470
	+	Dev	9	18	32	0.333	0.219	0.264
		Test	15	13	20	0.535	0.428	0.476
LR	-	Dev	17	7	24	0.708	0.414	0.523
		Test	17	6	18	0.739	0.485	0.586
	+	Dev	19	14	22	0.575	0.463	0.513
		Test	18	14	17	0.562	0.514	0.537
Ensemble	-	Dev	17	8	24	0.680	0.414	0.515
		Test	17	4	18	0.809	0.485	0.607
	+	Dev	21	17	20	0.552	0.512	0.531
		Test	20	13	15	0.606	0.571	0.588

For development and test sets, we employed the same development and test folders as in the segmenter stage.

As we report in Table 3, there are 41 CUs out of a total of 631 EDUs at development (0.0649 difficulty) and there are 35 CUs out of total 585 EDUs at testing (0.0598 difficulty). We use the development set for experimenting different alternatives.

All the evaluation results show the average performance of our classifier using recall (R), precision (P) and F-score obtained from the gold segmentation (Gold).

To evaluate human performance, in the first subsection of Table 7, we use average F-score of both annotators to compare the agreement of A1 and A2 annotators with respect to our super-annotator (gold CU), obtaining an F-score value of 0.634 at development and 0.849 at test set (0.215 over the development dataset).

The second subsection of Table 7 shows the BNB system (the best Basque CU detector) [56] that we used as our baseline. We can see that the BNB model does not get good results after adding 2 new domains (economy and computer science) to the system. We can confirm that the detection of the CU is heavily dependent on the domain when a CU is identified by keywords and some lexical-syntactic patterns. With respect to the performance of the BNB system on post-processing, the post-processing stage fails in all the decisions, but we included it when the CU detector needed to return at least one CU. In the case of BNB, the classifiers always return the probability of an EDU being labeled as CU. So, the post-process uses this value to select at least the most likely EDU to be labeled as CU.

The third subsection of Table 7 shows the 1-CNN results with pre-trained word embeddings. From our experiments, we observed that the ratio of “number of samples” (S) to “number of words per sample” (W) correlates with model performance. When the value for this ratio is smaller than 1,500, n-gram models, including Logistic Regression, Simple Multi-Layer Perceptron and SVM models (taking n-grams as input), perform better or at least as well as sequence models. When the value for this ratio is larger than 1,500, a sequence model such as CNN or Recurrent Neural Networks (RNN) is more suitable. In the case of our CU detector data, the samples/words-per-sample ratio is 169. The results shows that the 1-CNN system is the worst system, but could be helpful for enriching our ensemble system. The 1-CNN system with post-process obtained better results than without a post-process, attaining an F-score value of 0.264 at development. We stopped when error rate decreased at training while increasing at development. The total number of iterations was set to 23 in order to avoid over-fitting at training, resulting in an F-score value of 0.476 at test (0.041 less than our baseline).

The fourth subsection of Table 7 shows the LR with BoW, we see here that LR is the best simple model which provides 0.523 at development and 0.586 at test set. We find that LR is better than our baseline system, scoring 0.133 in the development and 0.022 in the test sets respectively. The results were worse when carrying out the post-process, while at development, the system succeeded in 2 decisions and failed in 7, at test set the system succeeded in 1 decision and failed in 8.

The fifth subsection in Table 7 presents our Ensemble unweighted voting system, in which, the class with the highest number of votes determines the prediction for the ensemble system. We can observe that this ensemble system is the best with and without post-process, obtaining 0.607 in F-score at test set without post-process, and 0.588 in F-score with it. This system is better than our baseline system by 0.125 in the development set and 0.043 in the test set without post-process, and 0.151 in the development set and 0.071 in the test set with post-process.

Secondly, we analyzed our systems using the segmenter’s output (Auto) tags. These systems were trained using the gold standard tags of segmentation, but tested using the segmentation tags (Auto) obtained from the Basque segmenter. To estimate the performance of our CU detector, the F-score value is estimated according to the exact-match scenario (we only take into account the segments that start with the same gold tag (B-SEG)). Table 8 shows the results of applying 4 different systems (BNB, 1-CNN, LR and Ensemble system) with and without post-process.

**Table 8:** CU results obtained from the segmenter’s output(Auto) with and without post-process stage.

System	Post	Data	C	E	M	P	R	F_1_
BNB	-	Dev	21	49	17	0.300	0.552	0.388
		Test	20	29	13	0.408	0.606	0.487
	+	Dev	21	49	17	0.300	0.552	0.388
		Test	20	29	13	0.408	0.606	0.487
1-CNN	-	Dev	8	4	30	0.666	0.210	0.320
		Test	13	5	20	0.722	0.393	0.509
	+	Dev	13	14	25	0.481	0.342	0.399
		Test	15	14	18	0.517	0.454	0.483
LR	-	Dev	15	6	23	0.714	0.394	0.508
		Test	16	6	17	0.727	0.484	0.581
	+	Dev	18	14	20	0.562	0.473	0.514
		Test	17	14	16	0.548	0.515	0.531
Ensemble	-	Dev	15	6	23	0.714	0.394	0.508
		Test	16	5	17	0.761	0.484	0.592
	+	Dev	18	12	20	0,600	0,473	0,529
		Test	19	15	14	0.558	0.575	0.567

We have obtained similar values using gold and auto tags at test set with all the systems. The best result is 0.592 at test with an ensemble system without post-process and 0.567 with post-process.

Finally, to check how well the method scales up, we have conducted a new experiment. Bearing in mind that the mean length of texts equals 20 segments and the longest text has 43 EDUs in the test set, we extracted the texts that have more than 20 segments. We applied the best system to those texts, that is, the ensemble system without post-process, obtaining 0.5 in F-score, 0.1 less than the value obtained using the whole set of test data(0.607 in F-score). Although there is a slight degradation (0.1), the detection of the CU seems to scale up properly to longer texts [43].

## 6 Discussion

### 6.1 Error analysis

#### 6.1.1 Segmentation error analysis

With the aim of understanding the output of the segmenter, we analyzed all the errors and we classified them taking into account the size and function of the discourse spans: *i*) complements (functioning as noun phrases) and relative clauses (functioning as noun modifiers), *ii*) non-finite adjunct clauses, *iii*) finite adjunct clauses, *iv*) independent clauses as part of the sentence, *v*) one sentence and *vi*) text spans from more than one sentence.

Until now the Basque segmenter [28] failed especially at intra-sentential EDUs (0.38 F-score), whereas the overall results were 0.73 F-score at test. In this work, we improved the overall results in 0.12 F-score at test set reducing the errors and low performance specially at the intra-sentential EDU detection.

However, as we can see in Table 9, there is still room for improvement at subordination intra-sentential level and also for the detection of other clause structures. For example, more that 50% of the errors occur in non-finite adjunct clauses and independent clauses. Most of the time, these are due to parsing errors such as wrong adjunct and coordinated clause detection, errors in the analysis of clauses with a strong discourse marker, parentheticals with verbs and list sentences. These kinds of sentences are hard to identify using the syntactic parser. Note that the corpus at hand lacks syntactic gold standard annotation and therefore we cannot offer the reader a quantitative evaluation of the parser’s errors over the whole test set. The strategy, then, has been to check whether the incorrectly segmented EDUs belonged to erroneously parsed sentences.

**Table 9:** Segmentation error analysis of undetected EDUs in the test set.

Function	Units	EDUs K	%
Subordination	Complement	11	12.94
	Non-finite adjunct	23	27.06
	Finite adjunct	9	10.59
Main clauses	Independent clause	28	32.94
	One sentence	8	9.41
	More than one sentence	6	7.06
**Total of EDUs**		85	100

As we stated above, in order to show the impact syntax and automatic POS information have on the segmenter, we employed the output of two different parsers as the input for our segmenter: i) Maltparser and ii) UDPipe parser. Segmentation using Maltparser achieved better results. Taking into account that Maltparser-based segmentation’s F-score improved by 0.9 on the development set and by 0.4 on the test set with respect to the segmentation based on UDPipe, this and the manual error analysis in this section highlight the impact syntax has on segmentation. Improving the results of the syntactic parser has a positive effect on the segmentation, because the segmenter uses syntactic tags as input. This leads us to think that if we had used MaltParser instead of UDPipe in the DISRPT 2019 Shared Task, our results would surely have been better.

#### 6.1.2 Central Unit detector error analysis

Regarding the CU detection, using the segmenter output, we manually checked the annotation results of the tool to describe the main errors of the system in the test set. To do so, we describe the four different types of agreement and a lack of agreement found in Table 10: *i*) **All CUs**. The system tag correctly identifies only the CU (or CUs, if the text contains multiple CUs) (Total agreement). *ii*) **Some CUs**. The system detected only one of the CUs without any error, but was not able to detect all the CUs, (Partial agreement). *iii*) **All CUs+EDUs**. All the CUs were detected, but the system also tagged other EDUs incorrectly as CUs, (Partial agreement). *iv*) **Not all CUs+EDUs**. The system detected a CU but not all of them and also incorrectly labeled EDUs as CUs, (Partial agreement). *v*) **Single EDUs**. The system detects other incorrect EDUs as CUs (No agreement).

**Table 10:** Relaxed error analysis results of CU detection of each text at test set.

	Agreement	Partial agreement			No agreement	No tag	Total
	All CUs	Some CUs	All CUs + EDUs	Not all CUs + EDUs	only EDUs (missed CUs)	No CU	Texts
Test	15	0	3	2	8	0	28
	53.57%	17.86%			28.57%	–	100%

Most of the times the CU is not declared or has few indicators, so it is difficult to detect it automatically. A reason for this can be, as [80] stated, that scholars have not had time to adapt “functionally to the situational context, nor to fix adequate linguistic patterns and formulaic sequences” to mark different discourse structures or, more specifically, to indicate the main aim or the Central Unit.

In a relaxed agreement 71.43% (20 of 28) of the documents in the test set the CU (or at least one of the CUs in multiple constructions) was tagged correctly (total and partial agreement). In 53.57% (15 of 28) of the documents, all CUs were correctly tagged (agreement in all CUs) and in 17.86% (5 of 28) were partially correctly tagged (CUs + EDUs). The system did not correctly tag 28.57% (8 of 28) documents.

We observed that the performance of the system varies depending on the dataset. The agreement between linguists was also very different in both datasets. The agreement of the annotators with respect to the gold standard was the following: in the development set, A1 agreed with 72.29% (F_1_) whereas A2 agreed with 55.00% (F_1_). In the test set, A1 obtained 90.14% (F_1_) agreement and A2 72.29% (F_1_).

The system detected all CUs in the texts belonging to economy, computer science and terminology domains, whereas it detected just some CUs in texts of life, medicine, health domains and it detected no CUs in the science domain. This fact needs further investigation to measure to what extent domain has an impact on the CU identification task. Although studying other kind of reasons such as writing style, journal conventions and language standarization level, might be very interesting, it is out of the scope of this work, because reaching significant conclusions regarding these issues would require larger annotated corpora than the ones we currently have.

Regarding the errors of the CU detector, the system failed for 13 texts. Here are some examples of these errors that show a better understanding of the task in our corpus. It is worth noting that sometimes the system could not identify CUs properly because the texts were poorly written.

- All CUs + EDUs: 3 cases. In these three cases the CU was not written correctly. An illustration of this point can be found in Example (2) the main aim of paper was not expressed explicitly in the first sentence (underlined). Besides, the second sentence (which is not the main topic of the paper) showed many more indicators. These two sentences were marked (in bold).
  (2) [Gure ikerketa taldearen lana prozesu hauen erregulazio peptidikoaren ezagutzan oinarritu da.]_*CU*_ (…) **Gure talde**ak beste ehun eta sistema fisiologiko batzuetan **garrantzia** daukaten **komunikazio** sistema **garrantzitsu** batzuk **aztertzen ari** da [BIZ19]
    ENGLISH TRANSLATION: [Our research team’s work has been based on the peptide regulation knowledge of these processes.]_*CU*_ **Our group** is **analyzing** an **important** **communication** systems of other physiological tissues and systems.
- Not all CUs + EDUs. There are two texts that do not follow the prototypical characteristics of the CUs. Example (3) shows a truncated EDU—ellipsis shows that there is a truncated EDU in the position—which is the CU of the text. As the segmenter does not link truncated EDUs, the CU detector could not detect this structure. Therefore the system only detected the first EDU.
  (3) Lan honen helburua (…) Bizkaiko baso-sektorearen egoera analizatzea eta bertako baso-politikan funtzio ekologikoek hartzen duten garrantzia aztertzea izan da. [EKO17]
    ENGLISH TRANSLATION: The objective of this work (…) is to analyze the situation of the forestry sector in Biscay and analyze the importance of the ecological functions in the forestry policy.
- Sufficient indicators that, however, where not detected by the system: 8 texts. Some CUs have multiple indicators but the system did not make use of them, such as in Example (4).
  (4) [Azken urteotan gure taldeak eritasun zeliakoaren genetika eta immunologia aztertu ditu hainbat ikuspuntu ezberdin eta berritzailetatik.]_*UZ*_ Bestalde duela zenbait urte **gure** laborategian egindako genoma osoko adierazpen azterketa bati esker eritasunean inplikaturiko hainbat bidezidor biologiko identifikatu eta sakonago **analizatu ditugu**. [*OSA*11]
    ENGLISH TRANSLATION: [Recently, our team has been studying genetic and immunology of celiac disease from several different and innovative perspectives.]_*UZ*_ On the other hand, **we have analyzed** and thoroughly identified several biological pathways involved in the disease through the analysis of a complete genome study in **our** laboratory a few years ago.

## 7 Conclusions and future work

This work presents an automatic tool based on neural networks that performs two tasks: *i*) segmentation and *ii*) detection of the CU. The system combines both tasks, outperforming previous work on CU detection [56] and achieving state-of-the-art results for segmentation [28].

Our initial aim was to obtain competitive segmentation results because this is the very first stage on the way to developing a complete parser and is the input for the Central Unit Detector. We implemented a neural-network-based segmentation which has proven to get better results than the previously employed rule-based system. Our system also equals state-of-the art results obtained with other systems.

One of the advantages of these networks is that they allow the use of word embeddings as input instead of the word strings themselves. These word embeddings are calculated in an unsupervised manner over large quantities of raw text. These vector representations enable better generalization because they are able to capture both syntactic and semantic information from the word itself. So, even though the size of the training corpus can still not be counted in millions of words, the embeddings in addition to the BiLSTM+CRF system helped to boost the results, affording an increase of around 30%.

This work also demonstrates the relevance of syntax and different word representations for accurate segmentation. A 20- and 14-F-score-point variation in the development and test set respectively, depending on the parser applied, and more than 9 and 3 F-score points at development and test respectively, depending on the different word representations selected, substantiate this conclusion.

On the top of that, we also tested different systems and features to detect the CU. We obtained the best results using the gold standard tags with an ensemble system with post-process which revealed an F-score of 0.588 at test set, outperforming the baseline system (the state of the art) by 0.071. Our best simple system with post-process is the Logistic Regression system with 0.537 F-score at test set. So we obtained an ensemble system which offers quality and diversity, with the following combination: BNB system with Linguistic Features (LF), 1-CNN with pre-trained word embeddings and a Logistic Regression model with BoW approach.

This work is the first of its kind to measure the impact on a Basque CU detector of using automatically obtained segments, in contrast to gold standard segments taken from the treebank. We used the segmenter output with different CU detectors: BNB with LF, 1-CNN with pre-trained word embeddings, LR with BoW and an Ensemble system. As a principal result, we can say that the errors due to the incorrect segmentation are not as important as we initially expected, as we obtained similar results across all the systems at test set. The best result is 0.592 at test set with an Ensemble system without post-process, and 0.567 with post-process.

Finally, we extended the corpus to the following domains: Economy and Computer science, outperforming the results, even though CU detection is domain oriented task.

For the future, results on NER and other seq2seq tasks have been substantially improved using contextualized word embeddings [81, 82] and framework [83] in recent experiments. This work showed us the effect different word representations have on the system, so the next step will be to test contextualized word embeddings as [47] did in DISRPT 2019 Shared Task.

We also plan to increase the size of the CU’s dataset to improve the results of CNN systems with pre-trained word embeddings.

In the short term, the authors are striving to implement a new module that identifies rhetorical relations linked to the CU, following a top down approach, and using our system for different tasks such as question answering [84], sentiment analysis [7] and summarization tasks [34].

This work can be easily adapted for other languages and domains, annotated with RST taken from the most prominent units in other sections or paragraphs of scientific articles or other kinds of texts.
